# Occupational Therapy for Adults with Overweight and Obesity: Mapping Interventions Involving Occupational Therapists

**DOI:** 10.1155/2018/7412686

**Published:** 2018-10-30

**Authors:** Svetlana Solgaard Nielsen, Jeanette Reffstrup Christensen

**Affiliations:** ^1^The Research Initiative of Activity Studies and Occupational Therapy, Research Unit of General Practice, Department of Public Health, The University of Southern Denmark, J.B. Winsløws Vej 9a, 5000 Odense C, Denmark; ^2^The Research Unit for Physical Activity and Health at Work, Department of Sports Science and Clinical Biomechanics, The University of Southern Denmark, Campusvej 55, 5230 Odense M, Denmark

## Abstract

**Background:**

Worldwide obesity rates are increasing. The effectiveness of occupational therapy in overweight and obese adults has not yet been clarified.

**Objectives:**

The scoping review aimed at examining the evidence on interventions involving occupational therapists in the treatment of adults with overweight and obesity.

**Methods:**

Data on interventions involving occupational therapists and reporting on lifestyle-related outcomes in overweight and obese adults was extracted from the databases Cochrane, PubMed, CINAHL, and Embase, including hand and reference search. The scoping review methodology of Arksey and O'Malley was used. Conclusions were based on numerical and narrative analysis.

**Results:**

Thirteen articles reporting on eleven studies met the inclusion criteria. Several studies showed significant weight loss. However, the studies possessed high heterogeneity and showed insufficient explication of the role and contribution of occupational therapy to the outcomes.

**Conclusions:**

The interventions with involvement of occupational therapists were suggested to help short-term weight loss. Occupational therapists contributed to the outcomes with a holistic approach, educating on the role of activity, providing technological support, and promoting enjoyment of being active. There is a need for further documentation of the effectiveness, role, and contributions of occupational therapy in the treatment of overweight and obese adults in all settings.

## 1. Introduction

The prevalence of obesity has increased continuously since 1980 and has even doubled in more than 70 countries [1]. Obesity relates to numerous health issues, such as cardiovascular disease, several types of cancer, and diabetes mellitus [2]. Besides resulting in health problems, obesity can impede engagement in meaningful daily activities and lower one's opportunities in education, leisure time, and work [3–5].

Lifestyle interventions, dieting, pharmacology, and bariatric surgery have been named as the methods typically used today in the treatment of adults with overweight and obesity [6–9]. The evidence has recommended combining a calorie-reduced diet (with the energy deficit of at least 500 kcal/day) and physical activity increased to 30 min in most weekdays, as the first-line option in obesity treatment [6–8]. An intervention ought to include behavioral treatment as the third component facilitating adherence to diet and physical activity recommendations, to be called “a lifestyle intervention” [6].

There is strong evidence for intensive lifestyle interventions which vary up to 6 months, for clinically significant weight loss (5–10% of initial weight, approximately 8 kg) [6]. There is moderate evidence for lifestyle interventions in intermediate-term weight loss (weight reduction with another 8 kg during the next 6 months of intervention) [6]. Although lifestyle interventions of duration ≥ 1 year have been associated with weight regain, they have shown a higher effect on weight loss compared to standard care, e.g., advice [6]. The interventions of high-frequency contacts with health professionals (≥14 contacts in total for the first 3 to 6 months) have achieved the best effect [6]. Single-component approaches have been found less effective than multicomponent approaches [6, 7]. The optimal duration of lifestyle interventions leading to clinically significant weight loss and optimal strategy for additional weight loss beyond the initial 6 months of intervention, as well as long-term approaches (2–5 years) to the maintenance of lost weight, are still to be clarified [6, 8, 10].

Researchers point at the need for all health professionals to be upskilled for effective management of the “obesity epidemic” [11, 12]. Occupational therapists possess key skills that help to promote health and to establish persistent lifestyle changes through participation in activities of choice, prevention of occupational deprivation, and increase in the perceived quality of life [3, 13]. However, the evidence of the effectiveness of occupational therapy in overweight and obese clients is scarce [14–17]. Several nonsystematic reviews have outlined the role, main target populations, domains, and strategies for occupational therapy addressing individuals with overweight and obesity [14, 17, 18]. Neither a systematic review nor a systematic investigation of the scope of occupational therapy interventions in the field has yet been conducted [14, 17, 18]. This study aimed at examining the evidence from studies evaluating the effectiveness of interventions involving occupational therapists in the treatment of adults with overweight and obesity.

## 2. Materials and Methods

### 2.1. Design

The current review followed Arksey and O'Malley's five steps of scoping review procedure with the advantage of methodological improvements done by Davis et al., Levac et al., Colquhoun et al., Tricco et al., and Peters et al. These are (1) defining the research question; (2) identifying relevant studies; (3) study selection and inclusion; (4) data charting; and (5) collating, summarising, and reporting of the results [19–25]. The 6th step of Arksey and O'Malley's scoping review procedure, consultation with stakeholders as a required knowledge translation component, will be reflected in Discussion. This study followed the principles of the Declaration of Helsinki [26].

### 2.2. Defining the Research Questions

- What characterizes the interventions involving occupational therapists identified in the current evidence?
- Which significant improvements in lifestyle and health behavior were made by adults with overweight and obesity who participated in interventions involving occupational therapists?

The search strategy with inclusion and exclusion criteria was developed using the PCC model (population, concept, and context) [24]. The three-fold focus in the search strategy was related to the following: adults with overweight or obesity, interventions that involved occupational therapists, and intervention outcomes showing changes in lifestyle and health behavior.

### 2.3. Identifying Relevant Studies

A three-step literature search was performed from February to April 2017 (last search: 22 April 2017) to identify studies that reported outcomes of interventions for adults with overweight or obesity, where occupational therapists were involved. Firstly, an initial literature search was made in PubMed to identify relevant keywords, synonyms, word modifications, and thesaurus terms, according to the PCC criteria in this study [24]. Secondly, the database-specific searches were conducted in the databases PubMed, Embase, CINAHL, and the Cochrane library using block search strategy. The thesaurus terminology of each database and words, e.g., “occupational therapy”, “occupational therapists”, “obesity”, “obese”, “overweight”, “bariatric”, “lifestyle”, “health behaviour”, “habits”, and “activities of daily living”, as well as their terminological variations, were included. Truncations were used when relevant. No time restrictions were used. Thirdly, additional publications of relevance were searched manually in reference lists. Google Scholar and Bibliotek.dk were inspected using the terms “occupational therapy” and “obesity” and “occupational therapy” and “overweight”. Unpublished items on interventions of interest were searched in ClinicalTrials.gov and WHO International Clinical Trials Registry Platform (ICTRP). Websites of organisations with expert knowledge in the field, the University of Southern California (USA), and Ergoterapeutforeningen (the Labour-Union for Occupational therapists in Denmark) were inspected. The second search was done in OTseeker on 22 April 2017. No further articles were found. The software reference program Endnote X8 was used to organise search results.

### 2.4. Study Selection and Inclusion

Selection of articles eligible for inclusion was guided by inclusion and exclusion criteria according to the research questions. The articles were selected in agreement with the authors. The inclusion criteria were as follows: (i) interventions in all settings addressing lifestyle in overweight or obese adults > 18 years; (ii) identified occupational therapists' involvement in the interventions; (iii) reported outcomes on the effectiveness of the interventions between participants before and after or between groups; and (iv) both articles published in peer-reviewed scientific journals and “grey literature,” e.g., treatment reports, evaluations, and public presentations. The exclusion criteria were as follows: (i) parents to children with overweight or obesity problems; (ii) pregnant women; (iii) articles written in languages other than English, Danish, Swedish, Norwegian, or German; and (iv) expert opinions, editorials, commentaries, interviews, conference thesis, lectures, periodicals, or abstracts.

**Figure 1:**
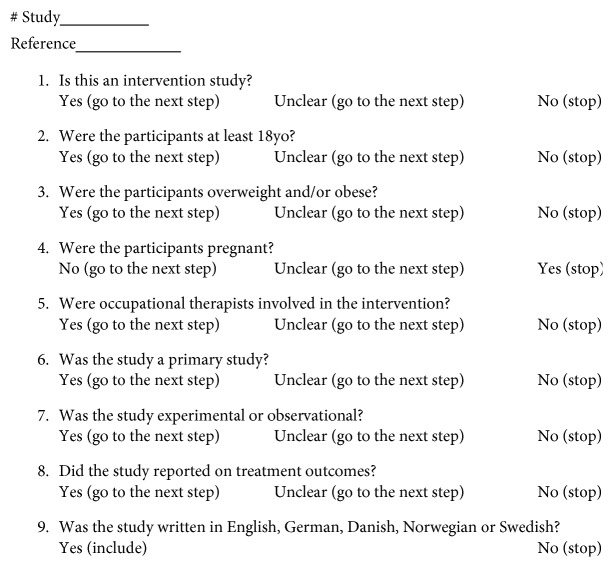
Selection form.

A selection form was developed to reduce the risk of selecting bias and support the iterative approach to the selection process [21] (Figure 1). The selection form assisted the decision-making process upon data screening. The articles that did not fit into the selection form were excluded. Both authors were involved in all parts of the review process. An agreement was achieved upon discussion between the authors.

### 2.5. Data Charting

The data charting form was developed and pilot-tested on a sample of three of the included articles in terms of further justifications. The final data charting form included information on the first author, year of publication, country of origin, publication source, study design, methods, sample size, the participants' age and gender, intervention's duration and content, comparator, and the role and contributions of occupational therapy to outcomes. Descriptive statistics on study results and effects at baseline, post intervention, and follow-up (when available) and *p* values were extracted and provided in the data extraction form.

### 2.6. Collating, Summarising, and Reporting the Results

Analysis of the extent, nature, and composition of the included studies was conducted. Infographics were applied to illustrate the results, supported by narrative comments. Nonnumerical findings were subject to qualitative thematic analysis. A tabular summary of the results across the reviewed studies was made in terms of mapping the evidence for answering the research question.

## 3. Results

**Figure 2:**
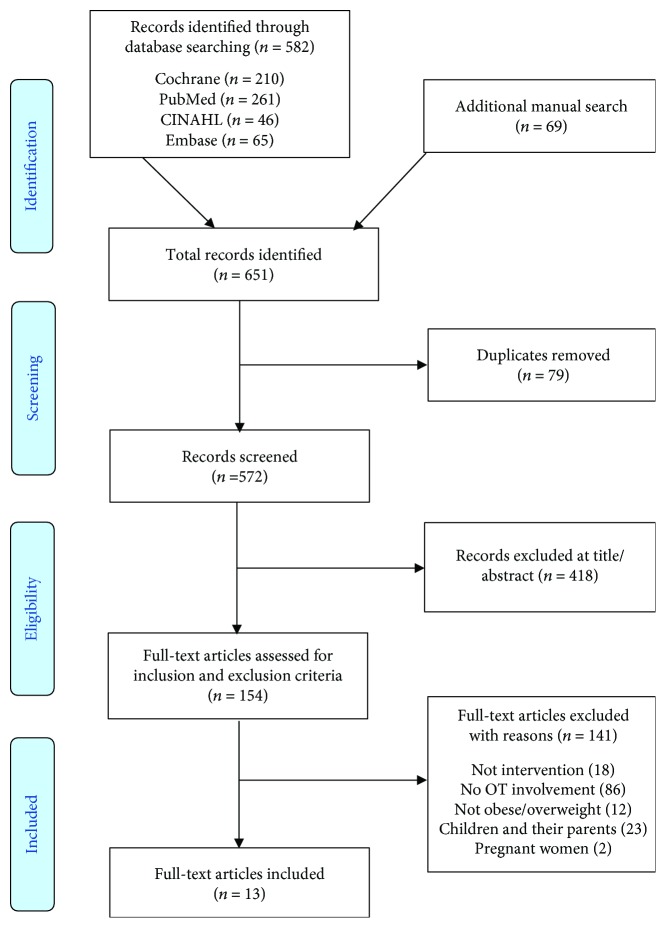
Flowchart, according to PRISMA [27].

The process for literature search, assessment, and selection is specified in the flowchart [27] (Figure 2). Initially, 582 records were sourced from the database search. Additional articles (*n* = 69) were found through reference lists (*n* = 651). After removing the duplicates (*n* = 79), the inclusion of the articles (*n* = 572) was made in two steps. All titles and abstracts were screened for relevance on the topic and excluded if they were not relevant for occupational therapy and obesity or overweight in the title, abstract, or keywords (*n* = 418). The remaining articles (*n* = 154) were inspected in full-text.

A total of 13 articles representing 11 studies describing interventions addressing adults with overweight and obesity, where occupational therapists were involved, were found eligible for this review.

The articles (*n* = 4) representing different phases of the same study (*n* = 2) were considered one study, in terms to avoid repetitive descriptions of the identical approach [28–31].

### 3.1. The Sample Characteristics

Three of the identified studies were RCT's [28–32], and three were quasiexperiments [33–35]. The rest (*n* = 5) were pre-/posttest studies of single cohorts [36–38] or single cases [39] or case groups [40] (Table 1).

**Figure 3:**
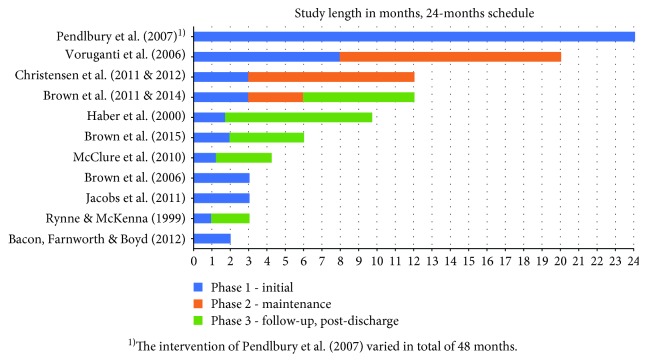
Interventions' phases and length.

Over the half of the included studies (*n* = 6) addressed individuals with mental problems (range 22–71 years old) [28, 29, 33–36, 39]. One study addressed diabetes patients (age range 37–87) [32] and another cancer patients (age range 42–79) [38]. Study samples of the participants who completed the interventions varied from 2 to 91 participants [28–31, 39, 40]. Many studies had high dropout rates 30–38% [28, 29, 32, 33, 37]. However, one study had only a few dropouts [30, 31], and another no dropout at all [35]. The interventions were composed of the following: 1-phase intervention (*n* = 4), 2-phase intervention (*n* = 6), and 3-phase intervention (*n* = 1) (Figure 3). Of the 2-phase intervention, two studies had an active maintenance phase that included ongoing treatment [30, 31, 35]. Thus, there were short-term (≤6 months of active treatment) (*n* = 8), intermediate-term (>6 months and ≤12 months of active treatment) (*n* = 1), and long-term weight loss interventions (>1 year of active treatment) (*n* = 2) in the sample [6].

The extent of occupational therapy involvement varied across the identified interventions. Two studies were solely occupational therapist-led [36, 39]. In another two studies, occupational therapists collaborated with other health professionals, either psychiatric nurses [34] or physical therapists [37]. The remaining studies (*n* = 7) were multidisciplinary interventions. The multidisciplinary profile in five studies included nurses, psychologists, dietitians, podiatrists, fitness instructors, sports scientists, or social workers [28–31, 33, 35, 38]. In two studies, the professionals involved, besides the occupational therapists, remained unspecified [32, 40]. The levels of transparency of occupational therapy engagement varied from reporting on the involvement of occupational therapists in several intervention processes, e.g., planning, execution, team supervision, and intervention management [28–31, 33–36, 38–40], to limited involvement, e.g., executing or team supervision only [32, 37].

Various attempts to promote the healthy lifestyle and health behavior changes in overweight and obese adults were described in the identified studies. Several studies from the sample did not declare any specific occupational therapy role and contribution. However, the studies operated with methods relevant to occupational therapy. Intervention components (as focus fields in an intervention) and intervention strategies (as methods of impact on the focus fields) across the studies were synthesized and differentiated according to the level of transparency in the declaration of the occupational therapy role and contribution (Table 2).

Regarding the major components of lifestyle interventions in obesity treatment described in the international guidelines in treatment of overweight and obesity, one-component (physical activity, *n* = 3) [35, 39, 40], two-component (physical activity and cognitive techniques, *n* = 1) [32], and three-component (diet, physical activity and CBT-elements, *n* = 7) [28–31, 33, 34, 36–38] studies were represented in the included articles.

### 3.2. The Reported Outcomes

All the studies aimed at making an impact on body weight in populations with obesity and/or risk of metabolic complications. Six studies (55%) were directly addressing weight change, while the rest focused on change in overall health behavior (*n* = 1) [37], self-management of disease symptoms (*n* = 2) [32, 38], or sedentary lifestyle (*n* = 2) [35, 39]. Weight loss, body mass index (BMI), and waist circumference were the most commonly used outcomes across the sample (Table 3).

#### 3.2.1. Weight Loss

Eight studies from the sample used weight loss to evaluate the intervention effect, and all found improvements [28–36, 40]. Significant body weight reductions were identified in short- [28–30, 32, 33, 36, 40], intermediate- [31], and long-term [34, 35]. The weight loss was most frequently achieved through a comprehensive approach combining physical activity, dieting and behavioral treatment [28–31, 33] compared to controls or in-group [36]. However, the combination of physical activity and behavioral treatment [32], as well as stand-alone physical activity or behavioral treatment [34, 35, 40], could also result in significant weight loss.

#### 3.2.2. BMI

BMI was assessed in five of the included in this research studies [30, 31, 33, 34, 38, 40]. Three studies identified a significant effect on BMI at intervention discharge [30, 33, 40], and one at the end of the maintenance phase [31]. Thus, one RCT found significant effects on BMI in short-term and intermediate-term [30, 31]. However, mixed results in different subgroups [40] and no significant results on BMI [38] were also found.

#### 3.2.3. Waist Circumference

A significant effect on waist circumference was experienced by the participants in two studies, one RCT and one quasiexperiment [30, 31, 33]. Waist circumference as an effect measure was chosen less frequently than changes in weight across the sample. The effects on waist circumference were maintained up to one year.

#### 3.2.4. Other Outcomes

Most studies used multiple outcome measures, such as a combination of objective anthropometric, biochemical, and physical variables and self-reported psychosocial variables. Both significant and nonsignificant findings were represented.

## 4. Discussion

The current study aimed at examining the evidence from studies evaluating the effectiveness of interventions involving occupational therapists in the treatment of adults with overweight and obesity. The most reviewed interventions were composed as multicomponent and multidisciplinary, involved graduated health professionals, offered frequent client contacts, and used elevated daily physical activity combined with better nutrition control, as recommended by the evidence on managing lifestyle changes in overweight and obese adults [11–14]. However, only seven interventions would fully match the definition of “comprehensive lifestyle interventions” having three components—physical activity, dieting, and cognitive behavioral therapy (or its elements) [11]. The sample did not sufficiently match the clinical recommendations to intervention length and reduction of energy intake, while the daily range of physical activity planned was not apparent [6, 8, 9].

### 4.1. Treatment Effects

#### 4.1.1. Weight Loss

Comprehensive lifestyle interventions having an impact on physical activity, diet, and behavior are recognized in other evidence as the most effective treatment aimed at weight loss in overweight and obese adults [6]. Overall evidence found three-component lifestyle interventions resulting in significant weight loss at the average follow-up of three years, with an average weight reduction of −2.2 kg [41]. Dieting in combination with physical activity brought better results in weight loss than physical activity alone [42, 43].

The current research found no significant improvement in weight from education as the only intervention form, which partly supports the importance of the comprehensive approach [38]. However, a number of interventions from the sample showing significant improvements in weight were not comprehensive per definition, as they had no dietary component included. The only intervention from the sample comparable in its duration (min. of 3 years) with the other evidence showed weight reduction above the average for lifestyle interventions in general [34].

The long-term (>1 year) effect on weight loss was found in two one-component interventions from the sample [34, 35]. They both had a high level of user-involvement and flexibility in planning, according to the participants' actual needs. We believe that the core principles of occupational therapy, such as client-centeredness and promotion of active participation, as well as the setting of realistic goals and using of meaningful occupations, might be the factors that allowed significant weight loss, despite less comprehensive intervention composition. As the two studies were both based on long-term contacts with occupational therapists and their collaborators, the results also supported the positive impact on weight loss of prolonged and frequent contacts with educated healthcare professionals [6, 7].

Only three interventions operated with the clinically significant weight loss measure (≥5% of the initial body weight) [28, 29, 34, 36]. Clinically significant weight loss was considered moderate and realistic to achieve, as well as being an important indicator for the satisfactory level of weight loss concerning human metabolic function and ability to prevent diabetes and hypertension [6, 7, 9]. Being aware of that would prevent unrealistic goals and underpin the favourable effects of weight loss starting with low weight loss levels [44]. Many other health science studies assessed clinically significant weight loss, and the parameter became an inclusion criterion for a systematic review on the topic [42].

The little focus on clinically significant weight loss in the identified interventions could be the consequence of the paucity in quantitative research on the topic, particularly RCTs, in the field of occupational therapy. We suppose that attention to clinically significant weight loss in occupational therapy interventions will become more common, as soon as further investigations of strong methodology emerge in the field, urging higher comparability of the results.

#### 4.1.2. Weight Regain

One study from the sample (an RCT with active treatment period = 6 months) showed nonsignificant weight regain in the intervention group at follow-up (12 months post recruitment/6 months post intervention) [29]. On the other hand, three interventions with active treatment duration ≥ 1 year were effective in the maintenance of the initial weight loss at the final assessment [30, 31, 34, 35]. The current research showed that sufficient weight maintenance can be achieved by 1-year continuous treatment, inclusive maintenance phase [30, 31].

Lifestyle interventions longer than 1 year were associated with weight regain [6]. Weight maintenance phases were recommended not to be ended earlier than ≥1 year from baseline [42]. However, maintenance phase duration > 1 year was not associated with a better effect on maintenance of the initial weight loss and its percentage [42]. Weight regain to preintervention level at 5 years post intervention was considered common for weight loss interventions and independent of BMI or metabolic status [7]. Weight regain could though be prevented by adapting individual weight maintenance strategies including continued healthy eating, high-level physical activity on regular basis, continued contacts with healthcare professionals (in any format), self-monitoring of body weight (e.g. once a week), and environmental support [7]. Additionally, maintenance of lost weight was found to require another approach, different from that for the initial weight loss [45].

While our findings supported the evidence, no interventions from the sample assessed the effect at 5 years post intervention. However, the two studies with the longest treatment durations (20–48 months) proved to achieve sufficient weight maintenance at the final assessment showing occupational therapy potentially capable of weight maintenance up to 4 years of treatment [34, 35]. The interventions were mainly based on either behavioral treatment or recreational outdoor physical activities.

From the above-named treatment elements important for weight maintenance, the two studies had their regularity, continued contact with occupational therapists, and environmental support (during the treatment sessions) in common. The presence of the other elements seemed more uncertain. Prolonged contacts with occupational therapists and supportive in-treatment environments might build up the sense of belonging through occupation in the participants and thus support weight maintenance after the initial weight loss. The positive correlation between belonging and well-being was found previously [46].

#### 4.1.3. BMI

BMI is a commonly used and recommended variable in weight loss interventions [6, 7]. However, the variable requires attention to possible assessment issues [7]. BMI may vary in different populations, because of differences in body fat and lean mass ratio depending on age, sex, race or nationality, or occupation, e.g., in athletes [47–49]. Other methods, e.g., measuring waist circumference, can be recommended to support BMI assessments in estimating the overweight and obesity burden on health [6].

#### 4.1.4. Waist Circumference

Similar to the sample studies, measuring of waist circumference was rather rare in other lifestyle interventions for adults with overweight and obesity [41]. However, lifestyle interventions may significantly reduce waist circumference compared to standard care, as well as maintain the effect for up to three years [41]. It is not known yet, whether the reported effects on waist circumference will sustain beyond one year of active treatment. Further investigations with at least three years of follow-up will also improve the comparability of occupational therapy results with other evidence on the reduction of waist circumference.

### 4.2. Occupational Therapy Role and Contribution to the Outcomes

As seen in the previous evidence, the identified interventions involving occupational therapists belonged to the secondary and tertiary health promotions, i.e., addressing adults in the risk of impairments or with present diagnoses [17, 50]. However, this review showed that the involvement of occupational therapists may also be relevant in primary health promotion of overweight and obesity, e.g., among healthcare workers and university students. As seen in the included interventions, occupational therapists appeared competent in the planning and execution of weight loss interventions, whether of mono- or multidisciplinary study setup. However, monodisciplinary occupational therapy interventions gave more space for an explication of the occupational therapy role and contributions to positive outcomes. However, we believe that multidisciplinary interventions involving occupational therapists offered a more specialized impact on lifestyle in overweight and obese adults as recommended in the international clinical recommendations. The occupational therapy impact declared in the included interventions did not include either education on nutrition and diabetes nor meal replacement. Since the topics on nutrition and disease may require specialized knowledge and skills, we found it appropriate that occupational therapists co-operated with dietitians, nurses, etc. in these fields. The multimodal and multidisciplinary approach to overweight and obesity has its advantages and is supported by evidence [51]. Thus, occupational therapy will consequently face the demand on an explication of its role, especially in multidisciplinary approaches.

We experienced that the current occupational therapy involvement was not comprehensively explicated and transparent in the reviewed interventions. The occupational therapy role and approach to treatment were reflected in a few articles from the sample. At the same time, the intervention components and strategies described in the articles with less transparency of occupational therapy involvement were close to those with clearly declared occupational therapy involvement, independently of mono- or multidisciplinary intervention character. Both types of interventions named above had similar components, e.g., physical activity practice, nutrition adjustments, relaxation techniques, cognitive techniques, and disease-specific elements. Both used collaborating with clients, education, setting individual goals, delivering instrumental, and social support, promoting active learning and sharing experiences, and supporting skill transfer to everyday life. However, education on the role of activity, focus on enjoyment from being active, and holistic approach to rehabilitation involving family and friends were only mentioned in the articles that delivered more comprehensive descriptions of occupational therapy contribution. Those qualities may be highlighted as the professional occupational therapy contribution in the interventions for overweight and obese adults. Additionally, occupational therapists contributed to the outcomes with a more rigorous use of VR (virtual reality) technology for exercise. Surprisingly, meal preparation and coping were only mentioned in the articles with no reports on a defined occupational therapy role. Meal preparation as a therapeutic tool would often be considered by occupational therapists in treatment planning [52]. Coping strategies, e.g., strengthening self-efficacy in an individual, would rather be in the occupational therapy scope as well [53]. We suppose that some core parts of the occupational therapy scope were lacking in this review due to the rather small sample size.

On the basis of the identified intervention components and strategies, all the reviewed studies could to a certain extent be linked to the previously outlined occupational therapy focus domains (e.g., “health promotion and prevention, increasing physical activity participation, modifying dietary intake, and reducing the impact of obesity”) and strategies (e.g., “assessment, modifying the environment, education, and introducing and adapting occupations”) [17]. All the interventions were promoting participation in adapted activities for weight loss to improve health and well-being and prevent disability [18]. However, the levels of adaptation and voluntary choice, as well as the scope of activities used within the interventions, varied across the sample. The fact of occupational therapists' involvement in the included studies contrasted with a poor specification of occupational therapy impact in the intervention descriptions. On the other hand, the similarities in the intervention components and strategies declared across the sample, including few monodisciplinary occupational therapy interventions, allowed us to suppose that occupational therapy impact in vivo might be greater than it was possible to detect in the current review.

The identified interventions link to occupational therapy also due to their focus on implementing of the new healthy lifestyle and sustainable changes in everyday practice related to physical activity, nutrition, and cognition, rather than only on weight-related outcomes. The evidence has described the occupational therapy role in lifestyle approaches as the mediator between some new wanted and needed healthy behaviors and an individual's habitual conditions [18]. Changing lifestyle and health behavior demands improvements in occupational performance through a holistic approach, which cannot be reduced solely to better physical fitness in an individual [54]. Thus, occupational therapy interventions may operate with a broader understanding of lifestyle, not limited to the presence of the three components (physical activity, nutrition, and cognitive treatment) mentioned in clinical recommendations to overweight and obesity treatment. We believe that every true occupational therapy intervention would potentially be “a lifestyle intervention” due to its focus on the whole person, knowledge transfer, and skills' adaptation into real life. Consultations with stakeholders, such as former and potential study participants, occupational therapy practitioners, other healthcare professionals from the multidisciplinary intervention teams, and researchers in the field of overweight and obesity may prove our assumptions and deepen the definition of the role and the impact of the OT in this area.

Occupational therapy contributed to the outcomes in the reviewed interventions with a holistic approach, sharing knowledge on the role of activity in people's life, supporting the new exercise routines with technology and encouraging enjoyment from being active. Further explication of the occupational therapy role and contribution in overweight and obesity treatment would deepen the understanding of occupational therapy potential in the field and let occupational therapists be involved in the future interventions for overweight and obese individuals at all levels of health promotion. For example, the use of assessment tools and indicators for changes in lifestyle and health behavior that are relevant for occupational therapy would open the door for more comprehensive descriptions of occupational therapy impact in future overweight and obesity interventions involving occupational therapists. We hope that the current review will inspire occupational therapy researchers to improve the quality and transparency of the evidence on the topic.

### 4.3. Study Limitations

Limitation of the methodological approach in this study is that scoping reviews provide an in-breadth overview on the topic, and not in-depth. This scoping review was not aimed to map all the literature on occupational therapy in the field of overweight and obesity but only focused on experimental studies from selected databases and with the identifiable involvement of occupational therapists. The selection strategy included keywords assigned by authors and may cause some of the relevant studies to be missing. Both primary and secondary articles usually are in focus of scoping reviews [55]. This review differentiated between these two categories, referring to the secondary evidence in the background and discussion sections of this study. Only primary publications were subject to analysis. Therefore, the scope of interventions addressing individuals with overweight and obesity and involving occupational therapists may be not accurately reflected in this scoping review.

## 5. Conclusion

The current review suggested that the interventions involving occupational therapists may help overweight and obese adults to achieve a significant change in weight loss in the short-term. Additional studies are still needed to confirm the suggestion. Whether occupational therapy can help the achievement of clinical significant intermediate- and long-term weight loss is still to be investigated.

This study found a little improvement in the evidence quality since Haracz et al. underscored insufficiency of the evidence in this field of research in 2013–14. A few randomized controlled blinded trials were identified in this study, which was indicating ongoing development in this area of practice and research. The review showed occupational therapists being competent actors in different parts of the intervention process in both the mono- and multidisciplinary overweight and obesity interventions. We found that occupational therapists contributed to the intervention outcomes with a holistic approach, providing knowledge on the role of activity in humans, supporting changes in health behavior with technology and promoting the enjoyment from being active.

We recommend the initiation of further comprehensive lifestyle interventions, e.g., randomized clinical trials, with the involvement of occupational therapists in the treatment of overweight and obese adults in all settings. The international clinical recommendations in the field, the OT-relevant assessment methods, and long-term follow-up phases ought to be considered for inclusion in the future interventions. Further evaluations of the effectiveness of the overweight and obesity interventions for adults involving occupational therapists together with a more comprehensive explication of the OT role and contributions to the intervention outcomes will improve the current evidence in this area.

## Figures and Tables

**Table 1:** Data extraction form.

Author (year), country [ref.], journal, purpose	Design, sample, age	Duration/frequency	Intervention/controls	OT role and contribution to outcomes	Outcomes	Results at discharge	Results at follow-up
Rynne & McKenna (1999), Australia [38] The British Journal of OT (BJOT), The Royal College of Occupational Therapists (UK) To evaluate an outpatient diabetes education program	Cohort One group Pre−/posttest Adults with non-insulin-dependent diabetes mellitus (*n* = 26) Females: 27% Mean age 74 yo (range 37–87 yo)	3 mths in total 1 mnth/1 hr á wk 2 mths follow-up	IG (participants and their relatives/friends) (groups at max. 10): information on the basic physiology of diabetes; management of hypoglycemia and sick days; medications and blood glucose testing; dietary management; weight control; role of PA; foot care; motivation; Diabetes Australia services	OT as part of multidisciplinary team Planning and executing, in line with a nurse, dietitian, psychologist, podiatrist and a representative of an NGO for diabetes Client-centered approach to the intervention; planning teaching-learning process; education on the role of activity and self-management of diabetes; training in managing weight control based on exercise recommendations from the national clinical guidelines (USA); supporting clients' self-management of lifestyle and adaptive behavior; systemic and holistic rehabilitation process; co-operation with community services	Diabetes knowledge	NS (unspec.)	NS (unspec.)
					Self-management behavior in diet	NS (unspec.)	NS (unspec.)
					Self-management behavior in exercise	(*p* < 0.001)^∗^	(*p* < 0.01)^∗^
					Perceptions of wellness	NS (unspec.)	NS (unspec.)
					BMI	NS (unspec.)	NS (unspec.)
					Metabolic control	NR	(*p* < 0.01)^∗^

Haber et al. (2000), USA [37] Family & Community Health journal, The Journal of Health Promotion and Maintenance (USA) To examine the impact of a health promotion program on the health behavior of older adults	Cohort One group Pre−/posttest Mixed-methods Older inactive, overweight and physically limited adults recruited from two sites IG included (*n* = 42) IG completed (*n* = 35) Mean age: 71 yo, range 64–89 yo Female 83%	9.75 mths in total 7 wks (=1.75 mths)/14 hr (1 hr twice a wk) Follow up: 8 mths post intervention	IG: 40 min PA; heart rate/PA intensity calculation; information on nutrition and stress management; 20 min group discussion on social, cognitive, and behavioral issues; social skills and environmental control training; realistic and measurable health goal setting; listing health benefits and motivational inspiration; self-affirmations; linking new health behavior with existing habits; homework assignments to increase PA time and healthy nutrition; phone calls between sessions	OT as part of OT/PT undergraduate team Executing, in co-operation with PT Active collaboration with clients; time administration; realistic and measurable health goals; supervised discussion in small groups; rethinking of existing habits, planning of new health behaviors, and environment control and modifications; adaptation of new exercise behavior; patient education and practice in progressive muscle relaxation; estimation of training intensity and heart-rate; individual diet calculation, in co-operation with dietitians; self-assessment on exercise and nutrition (additional fruit and vegetable consumption); information and experiential learning on stress management; using social support to motivation, listing health benefits and motivational inspiration, and repeating affirmations to oneself; improving memory function with social support	Brisk walk exercises	(*p* = 0.02)^∗^	NS (unspec.)
					Flexibility exercises	(*p* = 0.0001)^∗^	NS (unspec.)
					Strength exercises	(*p* = 0.01)^∗^	NS (unspec.)
					Association for health behavior change vs the following:		
					(i) Participants' educational level (ii) PT's involvement (iii) Participants' race	NR NR NR	NS (unspec.) NS (unspec.) NS (unspec.)
					Regular PA (min. of 3 t./wk at ≥20 min)	NR	NS (unspec.)

Voruganti et al. (2006) Canada [35] The Canadian Journal of Psychiatry (Canada) To assess the feasibility of clinical implementation and evaluate the effectiveness of a novel adventure- and recreation-based group intervention	Quasiexperiment Pilot, pre−/posttest Case–control Two groups Adults with schizophrenia IG (*n* = 23) CG (*n* = 31) Treatment adherence = 97%, no dropouts Mean ± SD age IG: 32 ± 7.5 yo Mean ± SD age CG: 41 ± 9.4 yo	20 mths in total 8 mths intervention (2 modules at 8 wks = 8 sessions) 12 mths of maintenance phase	IG: summer and winter modules with various outdoor activities. Participants encouraged to maintain weekly contacts with the treatment team between modules. CG: recruited from wait list, received standard clinical care included some recreational activities	OTs as part of multidisciplinary team Planning, executing, and supervising, in line with a nurse and a social worker No specific OT contributions declared OTs were involved in the multidisciplinary novel adventure-based intervention including outdoor activities for psychiatric rehabilitation			[Maintenance phase]
					Weight loss	NR	(*p* < 0.05)^∗^^a^
					Self-esteem	(*p* < 0.05)^∗^	(*p* < 0.05)^∗^
					Global functioning	(*p* < 0.05)^∗^	Marginally improved
					Self-appraised cognitive abilities	Marginally improved	NR

Brown et al. (2006), USA [33] Psychiatric Rehabilitation Journal, the American Psychological Association (USA) To examine the efficacy of psychiatric rehabilitation weight loss program	Quasiexperiment Two groups Pre−/posttest, pilot Adults with serious mental illnesses, BMI ≥ 25 Recruited (*n* = 59) Completed (*n* = 36) Dropout IG (*n* = 7); CG (*n* = 16) Completed IG (*n* = 21); CG (*n* = 15) Female IG: 71% Female CG: 60% Mean age IG: 47 yo, range 30–61 yo Mean age CG: 41 yo (range 30–61 yo)	3 mths in total 2 hr/wk	IG: weight loss and psychiatric rehabilitation principles; diet, frequent contact with professionals, dietary education, 30–45 min moderate PA 3–5 days/wk, goal setting, social and instrumental support, skill and transfer training (dining out), granted materials (calorie counts, cooking utensils etc.) CG: no treatment	OTs as part of multidisciplinary team Planning and executing, in line with a dietician and exercise psychologist No specific OT contributions declared OTs were represented in the multidisciplinary program aimed to utilize the psychiatric rehabilitation principles and weight loss strategies	Between-group diff.:		
					(i) Weight (ii) BMI (iii) Waist circumference (iv) Diastolic BP (v) Systolic BP (vi) Total lifestyle profile (vii) Lifestyle profile nutrition subscale (viii) Lifestyle profile PA subscale (ix) Energy intake	(*p* = 0.009)^∗^ (*p* = 0.008)^∗^ (*p* = 0.021)^∗^ (*p* = 0.82) (*p* = 0.23) (*p* = 0.51) (*p* = 0.35) (*p* = 0.037)^∗^ (*p* = 0.45)	
					In-group diff. (IG):	
					(i) Total lifestyle profile (ii) Lifestyle profile nutrition subscale (iii) Lifestyle profile PA subscale (iv) Energy intake (v) Fat intake	(*p* = 0.05)^∗^ (*p* = 0.023)^∗^ (*p* = 0.022)^∗^ (*p* = 0.045)^∗^ (*p* = 0.09)^∗^

Pendlebury et al. (2007), UK [34] International Journal of Neuropsychopharmacology (JNP), Oxford Academic (UK) To evaluate long-term changes in weight and patient attendance based on the outcomes from the first 4 years of a behavioral treatment program	Quasiexperiment Multiple treatment reversal designs, time-series, longitudinal Repeated pre−/posttest Adults with schizophrenia and affective disorder, on psychotropic medication, wishing to lose weight (*n* = 93) Total patient episodes (*n* = 103), incl. Reenrollments (*n* = 10) Females: 61% Age mean 43.7 ± 1.2 yo (range 22–71 yo)	4 years in total One session/wk	IG (open drop-in program): measuring weight; group discussion on dietary experiences; group discussion on 8 informal rotational topics (to solve any actual issues on weight loss)	OTs as part of multidisciplinary team Planning and executing, in line with a psychiatric nurse No specific OT contributions declared OTs were represented in the multimodal program that incorporated nutrition, exercise and behavioural intervention, providing a holistic lifestyle approach to weight loss	7% body weight and BMI change at 3–6–9 mths; 1–1, 5–2–3-4 y		
					Normal BMI achieved	23% [at the end of each patient episode]	NR
					Weight loss		Sign. NR^b^
					Weight loss correlation with young age	(*p* = 0.031)^∗^	NS (unspec.)
					Weight loss correlation with adherence to the program	(*p* < 0.0001)^∗^	NS (unspec.)
					Weight loss correlation with diagnosis	(*p* = 0.02)^∗^	NS (unspec.)
					Weight loss correlation with mono- or multimedication	NR	(*p* = 0.26)

McClure et al. (2010) USA [32] The American Journal of OT (AJOT), The American OT Association (USA) To report a randomised controlled study of a program, designed to achieve improvements in physical and emotional breast cancer–related lymphedema (BCRL) symptoms.	RCT Two groups Individuals with BCRL, BMI ≥ 29.8 (*n* = 32) IG (*n* = 16) CG (*n* = 16) Dropout (*n* = 11) Mean ± SD age IG: 57.0 ± 2.9 yo (30.7; 78.0) Mean ± SD age CG: 59.7 ± 2.1 yo (42.2; 78.7) Female: 100%	17 wks/4.25 mths in total 5 sessions at 2 hr/5 wks/1.25 mths and a self-monitored home program (3 mths)	IG (The Breast Cancer Recovery Program): of The FLOW video (McClure & Bittman, 2003) and relaxation techniques at home daily; verbal instructions and written educational material on lymphedema coping and relaxation techniques (deep diaphragmatic breathing, progressive muscle relaxation and facial massage); a question-and-answer component and group discussion at every session CG: professional advice/usual practice	OTs as part of multidisciplinary team (team composition not declared) Supervising the assessors; guiding the assessment process Mood and quality of life monitoring	Bio-impedance z (arm swelling)	(*p* = 0.049)^∗^	NR
					Arm flexibility	(*p* = 0.19)	(*p* = 0.10)
					Volume	NS (unspec.)	NS (unspec.)
					Weight loss	(*p* = 0.038)^∗^	Maintained sign. (unspec.)
					Quality of life in norm-based physical function	(*p* = 0.02)^∗^	NR
					Quality of life in general health	(*p* = 0.03)^∗^	NR
					Quality of life in vitality	(*p* = 0.05)^∗^	NR
					Mood	(*p* = 0.03)^∗^	(*p* = 0.017)^∗^

Jacobs et al. (2011), UK [40] British Journal of OT (BJOT), the Royal College of Occupational Therapists (UK) To investigate effect of Nintendo Wii Fit as an occupation to promote weight loss in students.	Cohort Three groups Pre−/posttest A-B design, explorative 1-year university students (*n* = 5) Dropout: 1 out of 6 Age> 18 yo Females: 100%	3 mths	IG 1: the solo Wii group (*n* = 2): individual Wii exercise, yoga, balance, and strength activities 4 t/wk, 30–45 min IG 2: the double Wii group (*n* = 2): the same training as group 2, but with a partner IG 3: the typical activity group (*n* = 1): moderate intensity physical activity (e.g., walking to class)	OTs as main interventionists Planning, executing Integral approach to PA, diet and activity participation; motivating for increase in PA; incorporating PA into daily routines; decreasing negative impact of obesity; promote participation in meaningful roles; using VR technology as a therapeutic tool for exercise; instructing in use of VR technology; encouraging to exercise with VR technology in leisure time	Weight:		
					(i) IG1 (ii) IG2 & IG3	(*r*^2^ = 0.53)^∗^ NS (unspec.)	
					BMI:		
					(i) IG1 (ii) IG2 & IG3	(*r*^2^ = 0.78)^∗^ NS (unspec.)	
					Motivation for PA:		
					(i) IG1 (ii) IG2 & IG3	NS, sugg. Improved NS (unspec.)	
					PA level:		
					(i) IG1 (ii) IG2 & IG3	NS, remained moderate NS (unspec.)	

Bacon et al. (2012), Australia [39] The British Journal of OT (BJOT), the Royal College of Occupational Therapists (UK) To evaluate the Nintendo Wii Fit use in changing engagement in PA	Pre-exp. explorative Single-case design Mixed methods Adults with mental illness (*n* = 2)	8 wks	IG: Wii Fit in individual or group sessions	OTs as main interventionists Planning, executing Providing access to meaningful PA PA participation on collaboration with the participants; positive role modelling; establishing positive activity behaviors and lifelong habits; instrumental support with VR technology for PA as part of the intervention and in leisure time; instruction in use of VR technology; making activity enjoyable	Total daily PA time	NS, increased	
						More positive attitudes towards PA	
					Attitudes towards PA		
						Increased PA, provided meaningful occupation and showed potential use of the technology	
					Use of Wii Fit		

Christensen et al. (2011), DK [30], and Christensen et al. (2012), DK [31] BMC Public Health (USA) To evaluate the effects of the first 3 mths and 12 mths of follow-up of a 1-year long lifestyle intervention aimed to achieve weight loss among health care workers	Cluster RCT To groups Single-blinded Overweight health care workers (*n* = 98) IG (*n* = 55) CG (*n* = 44) Females: 100% Divided into 7 groups Dropout phase 1 (*n* = 7) Dropout phase 2 (*n* = 8) Mean age 45.5 yo (range 36–55 yo)	12 mths in total 1 hr/wk during working time Two phases: weight loss-phase (3 mths), weight loss maintenance phase (9 mths)	IG: individually dietary plan with energy deficit of 1200 kcal/day (15 min/hr); strengthening exercises (15 min/hr) and CBT (30 min/hr); leisure time aerobic fitness: 2 hr/wk; additional reducing of energy intake; 15 min circuit training during the 6th–9th mth of intervention; local sport activities and jogging outdoor during the 9th–12th mth of intervention; motivation to use training log books for home exercises; composition of one's own diet; setting realistic easy-to-implement goals based on participants' preferences and perception of meaningfulness; coping with cravings and practicing the intervention principles in everyday life CG: monthly oral presentations at 2 hr during working time.	OTs as part of multidisciplinary team Planning/managing, executing, supervising No specific OT contributions declared OTs were represented in the multidisciplinary program that incorporated nutrition, exercise and behavioral intervention and applied to the clients' workplace and local environments			[Maintenance phase]
					Body weight	(*p* < 0.001)^∗^^c^	(*p* < 0.001)^∗^^d^
					BMI	(*p* < 0.001)^∗^	(*p* < 0.001)^∗^
					Body fat percentage	(*p* < 0.001)^∗^	(*p* < 0.001)^∗^
					Waist circumference	(*p* < 0.001)^∗^	(*p* < 0.001)^∗^
					BP	(*p* < 0.001)^∗^	(*p* < 0.001)^∗^
					Musculoskeletal pain	NS (unspec.)	NS (unspec.)
					Maximal oxygen uptake	NS (unspec.)	NS (unspec.)
					Isometric maximal muscle strength of 3 body regions	NS (unspec.)	NS (unspec.)

Brown et al. (2011), USA [28], and Brown et al. (2014), USA [29] Psychiatric Services, the American Psychiatric Association (USA)/Schizophrenia Research, the Schizophrenia International Research Society (USA) To access RENEW (recovering energy through nutrition and exercise for weight loss) program in individuals with serious mental illness at four mental health centers	RCT Two groups Adults with serious mental illness IG (*n* = 47) CG (*n* = 42) Enrolled (*n* = 136) Completed, at follow-up (*n* = 89) Females 61% Mean ± SD age 44.6 ± 10.9 yo	12 mths in total Intervention: 3 mths (3 hr/wk) Maintenance: 3 mths (3 hr/mth) Support: 6 mths	IG (RENEW): energy intake reduction min. 500 kcal/day; education on nutrition; PA min. of 30 min/day; individualized goal setting; eating together; 2 meal replacements a day; weekly phone support in maintenance phase, no contact in support phase CG: usual treatment (medication, case management, voluntary participation in day programs); no restrictions from attending wellness elsewhere	OTs as part of multidisciplinary team Planning, executing, in line with a nurse, dietician and fitness instructor No specific OT contributions declared OTs were represented in the multidisciplinary program that incorporated psychiatric rehabilitation principles and evidence-based weight loss strategies; providing education and practice in modifying nutrition and PA; incorporated social and instrumental support, goal setting, skills and transfer training, and cognitive compensation.	Weight loss 5% (clinically sign.) at 3 mths	(p = .01) ^∗e^	NR
					Weight loss 10% (weight loss maintenance) at 6 mths	(p = .22) ^f^	NR
					Weight regain at 12 months (follow up)		(p = .47) ^g^
					Differences by weight changes by site	At 3 vs 6 months: (p = .017) ^∗^ vs (p = .043) ^∗^	At 12 months: (p = .076)

Brown et al. (2015), USA [36] Psychiatric Rehabilitation Journal, The American Psychological Association (USA) To evaluate the Nutrition and Exercise for Wellness and Recovery (NEW-R) weight loss intervention.	Cohort Pilot pre−/posttest One group Adults with severe mental illness (*n* = 18) and a BMI> 25 Dropout: 2 out of 18 Females 89% Age range 23–64 yo Mean ± SD age 47.3 ± 10.5 yo	6 mths in total Intervention: 2 mths (16 hr, 2 hr./wk) Follow-up: 4 mths	IG: Education; PA (20–30 min. Moderate intensity); healthy meals; provided printed materials (recipes and books with guidelines for eating out) and exercise bands	OTs as main interventionists Planning, executing Increasing PA participation (moderate); practicing healthy eating in groups; instrumental support to healthy eating (recipes and guidelines for eating out) and exercise (elastic bands); encouraging positive cognition; motivating for sustainable health behavior changes in long term; planning daily behaviors with impact on weight; focusing on active learning and small changes	An average weight loss	(*p* = 0.12)^h^	(*p* = 0.03)^∗^^i^
					Increased knowledge about nutrition	(*p* = 0.05)^∗^	NR
					Increased PA	(*p* = 0.09)	NR
					Association between attendance and body weight	NS (but tended towards significance)	NR

BP = blood pressure; CBT = cognitive behavioral therapy; CG = control group; hr = hour; diff. = difference; IG = intervention group; *n* = number analysed; mth/mths = month/months; NGO = nongovernment organisation; NR = *p* value not registered; NS = not significant; OT = occupational therapy; OTs = occupational therapists; PA = physical activity; PTs = physical therapists; RCT = randomized controlled trial; sign. = significant; sugg. = suggested; unspec. = unspecified; VR = virtual reality; wk/wks = week/weeks; yo = years old. ^∗^ indicates statistically significant effect at 95% CI. ^a,b,c,d,e,f,g,h,i^ Mean weight loss in the intervention group: ^a^ −5.4 kg; ^b^ −6.2 kg; ^c^ −3.6 kg; ^d^ −5.8 kg; ^e^ −2.2 kg; ^f^ −1.9 kg; ^g^ −0.7 kg; ^h^ −1,4 kg; and ^i^ −4.3 kg.

**(a):** 

Intervention component categories	Intervention component modalities	Declared OT role in the sample [ref.]	The roles not specifically assigned to OT in the sample [ref.]
Physical activity (PA)	Interventionist-led	Promoting participation in moderate exercise [36]; assisting participation in exercise supported with VR technology [39, 40]	Promoting participation in PA [28, 29]; providing strengthening exercises at workplace, graduating PA progression [30, 31]; practicing PA in groups [33]; inclusion of feasible, available, assessable, affordable, and likely effective outdoor activities that are time-limited and suitable for evaluation, replication, and implementation into mental health services [35]
	In leisure time/self-managed	Providing access to exercise, e.g., with elastic bands [36] and VR technology [39, 40]; encouraging behavior changes by self-assessment of PA [37]	Encouraging continued strengthening exercises and initiating aerobic exercises at home [30, 31]

Relaxation techniques	Interventionist-led	Practicing progressive muscle relaxation [37]	Use of relaxation techniques [32]
	In leisure time		Encouraging home relaxation practice [32]

Nutrition	Dieting	Encouraging behavior changes by self-assessment of additional fruit and vegetable consumption [37]	Encouraging calorie reduction [28, 29]; composing individual dietary plan based on the Danish dietary recommendations, dietary records, and identification of dietary preferences, using evidence-based guidelines for calorie reductions [30, 31]; using recommendations from the clinical guidelines in treatment of overweight and obesity in adults (USA) and encouraging proper fluid intake [33]
	Meal replacement and meal preparation		In combination with identification of food preferences and ideas to preparation of favorite foods in a healthy way, moving from meal replacement to purchasing food at the grocery store [28, 29]; teaching to move from meal replacements to purchasing food at the grocery store, improving food purchasing habits and meal preparation techniques minimizing the need for extensive menu planning and cooking [33]
	Social eating	Providing healthy meal experience as part of group sessions [36]	Providing experiences in eating together [28, 29] and dining out [33]

Cognitive techniques	CBT elements	Encouraging positive cognition [36]	Using CBT elements in promoting health behavior changes at workplace, encouraging positive thinking [30, 31]
	Coping		Reflecting dysfunctional attitudes and coping behaviors [30, 31]; instructing in coping techniques [32]
	Memory support	Guidance in improving memory function with social support [37]	Teaching compensatory strategies for cognitive impairments [28, 29], i.e., as part of psychiatric rehabilitation strategies [33]
	Motivational support	Guidance in using social support to motivation, listing health benefits and motivational inspiration, repeating affirmations to oneself, and environment modifications [37]; making activity enjoyable [39]; positive role modelling [39]	Using simplification of material, active learning, repetition, flexible methods of presenting information, visual aids and reinforces [33]; improvement of motivation, self-esteem, and sense of belonging [35]

Disease-specific topics		Mood and quality of life monitoring in postsurgical breast cancer survivors [32]	Diabetes management in relation to hypoglycemia, sick days, medication, blood glucose testing, foot care, and psychological issues [38]

**(b):** 

Intervention strategies	Intervention strategy modalities	Declared OT role in the sample [ref.]	The roles not specifically assigned to OT in the sample [ref.]
Assessment		Supervising the assessors and guiding the assessment process [32]	

Education	On nutrition		Instructing in nutrition [28, 29]; teaching the importance of regular eating [34]; teaching identification of energy values, use of food labels, food composition, and appropriate portion sizes, with focus on experiential learning [33]
	On exercise	Providing exercise recommendations based on clinical guidelines (USA) within a multidisciplinary intervention [38]	Recommending moderate PA 3–5 times a week [33]
	On the role of activity	Education on the role of activity [38]	Teaching the importance of daily activity scheduling [34]
	On disease		Teaching self-management of diabetes [38]
	On stress management	Providing information and experiential learning on stress management [37]	
	Unspecified	Having focus on active learning [36]	Providing information on various rotational topics in relation to healthy lifestyle [34]

Individual goal setting		Promoting individual choice and assistance in setting daily and weekly goals [36]; helping in setting realistic and measurable goals [37]	Help in setting individualized goals [28, 29, 33], i.e., individual weight loss goals [30, 31]

Group discussion	Interventionist-led	Providing supervised discussion in small groups [37]	Building up team spirit to prevent dropout [30, 31]; promoting of sharing experiences, question-and-answer approach for providing modified learning opportunities for an individual [32]; providing social support through group interaction [33]; encouraging patients to help each other through voluntary experience exchange [34]

Phone call support		Providing encouragement and support to health behavior changes [37]	Weekly phone calls during maintenance phase aimed problem solving and goal setting, monthly phone calls in follow-up phase to promote sustainability [28, 29]

Instrumental support	Printed/written materials	Supporting behavioral changes with recipes and guidelines for eating out [36]	Providing disease-related printed materials [32]; weekly newsletter in maintenance phase monthly mails in follow-up phase with tips and reminders encouraging healthy lifestyle [28, 29]; promoting calorie count guides [33]
	Video guiding		Video guide for self-monitoring of disease-related health issues in breast cancer survivors [32]
	Exercise tools	Promoting accessibility to exercise through providing elastic bands [36]; supporting exercise with VR technology [39, 40]	Providing training tools, e.g., pedometers, weights, stretch bands, heart rate monitors, and workout videotapes [33]
	Cooking utensils		Providing cooking utensils to promote proper nutrition [33]
	Unspecified		Instrumental support given/unclear [28, 29]

Skill training	Weight control	Training in managing weight control [38]	
	Exercise self-management	Teaching to estimate own training intensity and heart-rate [37]	
	Relaxation techniques	Teaching progressive muscle relaxation [37]	Instruction in relaxation techniques [32]
	One's own diet composition	Co-operating with dietitians in helping clients to calculate an individual diet [37]	
	Use of technology	Instructing in use of VR technology in exercise [39, 40]	
	Self-control for sustainable health behavior changes	Planning daily behaviors that can impact weight with focus on small changes [36]; inspiration for rethinking of existing habits, planning of new health behaviors and environment control [37]	Focusing on transferring behavioral changes into habit patterns in maintenance phase, identifying small successes and issues in daily living [28, 29]; Using a fast food guide on a dining out session [33]
	Social skills		Improvement of social skills [35]

Homework assignments	On exercises	Encouraging behavior changes by self-assessment [37]	Encouraging positive thinking with homework between sessions [30, 31]; daily PA log [33]
	On nutrition	Encouraging behavior changes by self-assessment [37]	Nutrition log [33]

Community involvement	Patient organisation	Promoting co-operation with community services [38]	Co-operating with a community support program to provide support between group sessions [33]
	Family and friends	Prompting systemic and holistic rehabilitation process [38]	
	Local sport and leisure facilities		Encouraging using local sport facilities to increase daily PA [30, 31]; planning and promoting participation in adventure outdoors activities [35]

BP = blood pressure; CBT = cognitive behavioral therapy; OT = occupational therapy; PA = physical activity.

**Table 3:** Summary of the reported outcomes.

Outcomes reported		Significant at discharge, studies (*n*)	Significant at follow-up, studies (*n*)	Nonsignificant at discharge, studies (*n*)	Nonsignificant at follow-up, studies (*n*)
Anthropometrics	Weight loss	6	4	3	1
	Weight regain	—	—	—	1
	BMI	3	1	3	1
	Body fat percentage	1	1	—	—
	Waist circumference	2	1	—	—

Biochemical and physical	Blood pressure	1	1	1	—
	Metabolic control measure	—	1	—	—
	Max oxygen uptake	—	—	1	1
	Isometric max muscle strength	—	—	1	1
	Flexibility, arm	—	—	1	1
	Bio-impedance z (arm swelling)	1	—	—	—
	Increased physical activity (alone or in small, or bigger groups)	—	—	3	1
	Brisk walk	1	—	—	1
	Flexibility	1	—	—	1
	Strength	1	—	—	1
	Lifestyle profile, physical activity subscale (between groups)	1	—	—	—
	Lifestyle profile, physical activity subscale (in-group)	1	—	—	—
	Lifestyle profile, nutrition subscale (in-group)	1	—	—	—
	Lifestyle profile, nutrition subscale (between groups)	—	—	1	—
	Musculoskeletal pain	—	—	1	1

Psychosocial	Global functioning	1	—	—	1
	Quality of life, in norm-based physical function	1	—	—	—
	Quality of life, in general health	1	—	—	—
	Quality of life, in vitality	1	—	—	—
	Mood	1	1	—	—
	Motivation	—	—	1	—
	Self-esteem	1	1	—	—
	Perception of wellness	—	—	1	1
	Self-management behavior in exercise	1	1	—	—
	Attitudes towards exercise	—	—	1	—
	Increased knowledge about nutrition	1	—	—	—
	Energy intake (in-group)	1	—	—	—
	Energy intake (between groups)	—	—	1	—
	Fat intake (in-group)	1	—	—	—
	Self-management behavior in diet	—	—	1	1
	Diabetes knowledge	—	—	1	1
	Differences by weight changes by site	1	—	—	1

Not identified outcome reports are marked with “—.”

## References

[1] GBD 2015 Obesity Collaborators, Afshin A., Forouzanfar M. H. (2017). Health effects of overweight and obesity in 195 countries over 25 years. *The New England journal of medicine*.

[2] World Health Organization Obesity and overweight, fact sheet. http://www.who.int/mediacentre/factsheets/fs311/en/.

[3] Clark F., Reingold F. S., Salles-Jordan K. (2007). Obesity and occupational therapy (position paper). *The American Journal of Occupational Therapy*.

[4] Ilvig P. M., Christensen J. R. (2017). Degree of disability among female healthcare workers who are overweight or obese. *Cogent Medicine*.

[5] Wang S. S., Brownell K. D., Wadden T. A. (2004). The influence of the stigma of obesity on overweight individuals. *International journal of obesity and related metabolic disorders*.

[6] National Health, Lung, and Blood Institute (2013). *Managing overweight and obesity in adults: Systematic evidence review from the Obesity expert panel. Evidence report*.

[7] National Health and Medical Research Council (2013). *Clinical Practice Guidelines for the Management of Overweight and Obesity in Adults, Adolescents and Children in Australia*.

[8] Lau D. C., Douketis J. D., Morrison K. M. (2007). 2006 Canadian clinical practice guidelines on the management and prevention of obesity in adults and children [summary]. *CMAJ*.

[9] Yumuk V., Tsigos C., Fried M. (2015). European guidelines for obesity management in adults. *Obesity Facts*.

[10] The Danish Health Authority (2018). *Overweight – The Prevention Pack*.

[11] James W. P. (2008). WHO recognition of the global obesity epidemic. *International Journal of Obesity*.

[12] Lang J., James C., Ashby S. (2013). The provision of weight management advice: an investigation into occupational therapy practice. *Australian Occupational Therapy Journal*.

[13] Forhan M. (2008). Obesity and healthy occupations. *Discussion Document*.

[14] Forhan M., Gill S. (2013). Cross-border contributions to obesity research and interventions: a review of Canadian and American occupational therapy contributions. *Occupational Therapy In Health Care*.

[15] Forhan M. (2014). Weight loss interventions for rehabilitation patients with obesity. *Current obesity reports*.

[16] Heslop J. (2009). A critical review into the role of occupational therapy in promoting the quality of life for people with bariatric needs. *British Journal of Occupational Therapy*.

[17] Haracz K., Ryan S., Hazelton M., James C. (2013). Occupational therapy and obesity: an integrative literature review. *Australian Occupational Therapy Journal*.

[18] Reingold F. S., Jordan K. (2013). Obesity and occupational therapy. *The American Journal of Occupational Therapy*.

[19] Arksey H., O'Malley L. (2005). Scoping studies: towards a methodological framework. *International journal of social research methodology*.

[20] Davis K., Drey N., Gould D. (2009). What are scoping studies? A review of the nursing literature. *International journal of nursing studies*.

[21] Levac D., Colquhoun H., O'Brien K. K. (2010). Scoping studies: advancing the methodology. *Implementation Science*.

[22] Colquhoun H. L., Levac D., O'Brien K. K. (2014). Scoping reviews: time for clarity in definition, methods, and reporting. *Journal of Clinical Epidemiology*.

[23] Tricco A. C., Lillie E., Zarin W. (2016). A scoping review on the conduct and reporting of scoping reviews. *BMC Medical Research Methodology*.

[24] Peters M. D., Godfrey C. M., Khalil H., McInerney P., Parker D., Soares C. B. (2015). Guidance for conducting systematic scoping reviews. *International journal of evidence-based healthcare*.

[25] Khalil H., Peters M., Godfrey C. M., McInerney P., Soares C. B., Parker D. (2016). An evidence-based approach to scoping reviews. *Worldviews on evidence-based nursing.*.

[26] World Medical Association (2013). Declaration of Helsinki: ethical principles for medical research involving human subjects. *JAMA*.

[27] Moher D., Liberati A., Tetzlaff J., Altman D. G., The PRISMA Group (2009). Preferred reporting items for systematic reviews and meta-analyses: The PRISMA Statement. *PLoS medicine*.

[28] Brown C., Goetz J., Hamera E. (2011). Weight loss intervention for people with serious mental illness: a randomized controlled trial of the RENEW program. *Psychiatric services*.

[29] Brown C., Goetz J., Hamera E., Gajewski B. (2014). Treatment response to the RENEW weight loss intervention in schizophrenia: impact of intervention setting. *Scizophrenia Research*.

[30] Christensen J., Faber A., Ekner D., Overgaard K., Holtermann A., Søgaard K. (2011). Diet, physical exercise and cognitive behavioral training as a combined workplace based intervention to reduce body weight and increase physical capacity in health care workers - a randomized controlled trial. *BMC Public Health*.

[31] Christensen J. R., Overgaard K., Carneiro I. G., Holtermann A., Søgaard K. (2012). Weight loss among female health care workers - a 1-year workplace based randomized controlled trial in the FINALE-health study. *BMC Public Health*.

[32] McClure M. K., McClure R. J., Day R., Brufsky A. M. (2010). Randomized controlled trial of the breast cancer recovery program for women with breast cancer–related lymphedema. *The American Journal of Occupational Therapy*.

[33] Brown C., Goetz J., Van Sciver A., Sullivan D., Hamera E. (2006). A psychiatric rehabilitation approach to weight loss. *Psychiatric Rehabilitation Journal*.

[34] Pendlebury J., Holt R., Wildgust H., Holt R. I. G. (2007). Long-term maintenance of weight loss in patients with severe mental illness through a behavioural treatment programme in the UK. *Acta Psychiatrica Scandinavica*.

[35] Voruganti L., Whatham J., Bard E. (2006). Going beyond: an adventure- and recreation-based group intervention promotes well-being and weight loss in schizophrenia. *The Canadian Journal of Psychiatry*.

[36] Brown C., Read H., Stanton M., Zeeb M., Jonikas J. A., Cook J. A. (2015). A pilot study of the nutrition and exercise for wellness and recovery (NEW-R): a weight loss program for individuals with serious mental illnesses. *Psychiatric Rehabilitation Journal*.

[37] Haber D., Looney C., Babola K., Hinman M., Utsey C. J. (2000). Impact of a health promotion course on inactive, overweight, or physically limited older adults. *Family & Community Health*.

[38] Rynne A., McKenna K. (1999). Evaluation of an outpatient diabetes education programme. *British Journal of Occupational Therapy*.

[39] Bacon N., Farnworth L., Boyd R. (2012). The use of the Wii fit in forensic mental health: exercise for people at risk of obesity. *British Journal of Occupational Therapy*.

[40] Jacobs K., Zhu L., Dawes M. (2011). Wii health: a preliminary study of the health and wellness benefits of Wii fit on university students. *British Journal of Occupational Therapy*.

[41] Galani C., Schneider H. (2007). Prevention and treatment of obesity with lifestyle interventions: review and meta-analysis. *International Journal of Public Health*.

[42] Ramage S., Farmer A., Eccles K., McCargar L. (2014). Healthy strategies for successful weight loss and weight maintenance: a systematic review. *Applied Physiology, Nutrition, and Metabolism*.

[43] Batsis J., Gill L. E., Masutani R. K. (2017). Weight loss interventions in older adults with obesity: a systematic review of randomized controlled trials since 2005. *Journal of the American Geriatrics Society*.

[44] Womble L., Wang S., Sarwer D. (2000). Do patients adjust weight loss expectations?. *Obesity Research*.

[45] Sciamanna C., Kiernan M., Rolls B. J. (2011). Practices associated with weight loss versus weight-loss maintenance: results of a national survey. *American Journal of Preventive Medicine*.

[46] Whalley Hammell K. R. (2014). Belonging, occupation, and human well-being: an exploration: Appartenance, occupation et bien-être humain: Une étude exploratoire. *Canadian Journal of Occupational Therapy*.

[47] Bambrick H. (2005). Relationships between BMI, waist circumference, hypertension and fasting glucose: rethinking risk factors in indigenous diabetes. *Australian Indigenous Health Bulletin*.

[48] Deurenberg P., Deurenberg-Yap M., Guricci S. (2002). Asians are different from Caucasians and from each other in their body mass index/body fat per cent relationship. *Obesity Reviews*.

[49] James W., Jackson-Leach R., NiMhurchu C., Ezzati M., Lopez A., Rodgers A., Murray C. (2004). Overweight and obesity (high body mass index). *Comparative Quantification of Health Risks: Global and Regional Burden of Disease Attributable to Selected Major Risk Factors*.

[50] Scriven A., Atwal A. (2004). Occupational therapists as primary health promoters: opportunities and barriers. *British Journal of Occupational Therapy*.

[51] Brewster K. Z., Nowrouzi B., Davis L. (2014). The role of occupational therapy in obesity management. *University of Toronto Medical Journal*.

[52] Bryant W., McKay E. (2005). What's cooking? Theory and practice in the kitchen. *British Journal of Occupational Therapy*.

[53] Gage M. (1992). The appraisal model of coping: an assessment and intervention model for occupational therapy. *The American journal of occupational therapy*.

[54] Orellano E., Colón W. I., Arbesman M. (2012). Effect of occupation- and activity-based interventions on instrumental activities of daily living performance among community-dwelling older adults: a systematic review. *The American journal of occupational therapy*.

[55] Grimshaw J., McAuley L., Bero L. (2003). Systematic reviews of the effectiveness of quality improvement strategies and programmes. *Quality & safety in health care*.

